# Hysteroscopy as a minimally invasive surgery, a good substitute for invasive gynecological procedures

**Published:** 2012-07

**Authors:** Seddigheh Abdollahi Fard, Parvin Mostafa Gharabaghi, Farnaz Montazeri, Omid Mashrabi

**Affiliations:** 1 *Department of Obstetrics and Gynecology, Women's Reproductive Health Research Center, Tabriz University of Medical Sciences, Tabriz, Iran.*; 2 *Department of Obstetrics and Gynecology, Faculty of Medicine, Tabriz University of Medical Sciences, Tabriz, Iran.*; 3 *Faculty of Medicine, Tabriz University of Medical Sciences, Tabriz, Iran.*

**Keywords:** *Hysteroscopy*, *Abortion*, *Infertility*, *Uterine bleeding*

## Abstract

**Background: **Hysteroscopy is a safe and high efficient procedure so it is changing to a widespread procedure in dealing with many gynecologic and obstetrical conditions.

**Objective: **This study aimed to evaluate the diagnostic and therapeutical efficiency of hysteroscopy in managing the common conditions including abnormal uterine bleeding, abortion and infertility.

**Materials and Methods:** This was a descriptive cross-sectional study to compare hysteroscopy as a minimally invasive approach with conventional laparatomy and hysterectomy or repair of mulerian anomalies and watch the uterine cavity for intrauterine pathology in cases of infertility. Overall 277 women underwent hysteroscopy were evaluated in three groups: with AUB 226 cases, with infertility 34 cases and with recurrent abortions with septate uterus17 cases. The overall success rate was recorded and analyzed after six months in order of indication of hysteroscopy

**Results:** Hysteroscopy as sole diagnostic procedure in 16.5, 8.8 and 14.3%of AUB, infertility and abortion cases, respectively. In AUB cases, curettage, myomectomy, polypectomy and hysterectomy were the main diagnostic-therapeutical approaches along with hysteroscopy. In infertiles, myomectomy, polypectomy were the main diagnostic-therapeutical approaches

In abortion group, laparoscopy guided, septum resection adhessiolysis , curettage and myomectomy were the main aproach. There was not any major complication. The diagnostic-therapeutically measures accompanying with the hysteroscopy were successful in 73.5% of the bleeding group and 33.3% of the infertility group in follow-up period.

**Conclusion:** Based on our results, hysteroscopy is a safe, accurate and highly-efficient procedure in managing women with abnormal uterine bleeding, recurrent abortion due to septate uterus

## Introduction

Hysteroscopy is a method through which endometrial cavity can be observed and manipulated transcervically. “Hysteroscopy is considered a minimally invasive approach which can be used for data analysis and treatment of numerous intrauterine and endocervical problems (1)”. 

“In many developed countries, hysteroscopy has replaced curettage in diagnosing and if possible treating abnormal uterine bleeding sources. The advantage of this method is the direct view and simultaneous intervention (2-4)”. Although hysteroscopy is not considered a common portion of infertility study process, it can be used to evaluate uterine effectively compared to hysterosalpingography (, 5). “Mullerian system abnormalities are the most common intrauterine disorders which are associated with infertility problems and spontaneous abortion. These disorders are related to developmental defects (agenesis, unicorn uterus), junction defects (bicornuate uterus) or defects in m tube canalization (septated uterus) (6)”. 

“Primary diagnosis is difficult due to the wide range of symptoms in these disorders including menstruation bleeding occlusion in the beginning of puberty, hypermenorrhea, vaginal discharges, dyspareunia, infertility and abortion (2)”. Diagnostic measurements are based on hysterosalpingography, laparoscopy and hysterosonography. Many of the disorders can be observed primarily in hysterosalpingo-graphy and sonogram but to be approved further imaging is a required. In these cases, MRI is considered the first option. Laparoscopy and hysteroscopy are kept for the patients needing interventional treatments (7). 

Considering the efficacy and safety of hysteroscopy in diagnosing and treating many common obstetrical and gynecological conditions, expanding this method in our centers is an essential; a fact that has not been focused on so far. Therefore, in this study we are to evaluate the diagnostic and therapeutic efficacy of hysteroscopy in the common positions like abnormal uterine bleeding, infertility and recurrent abortion.

## Materials and methods

In a descriptive cross sectional study with the aim of evaluating and replacing the hysteroscopy as a minimally invasive procedure in gynecological most common complaints, with conventional surgical procedures, 277 patients in reproductive ages who were admitted with abnormal uterine bleeding (226 cases), infertility (34 cases) and the repetitive abortion (17 cases) were studied in three groups.

Underlying reasons of abnormal uterine bleeding, infertility and abortion were clarified in hysteroscopy and their frequency in each group was determined based on the treatment type. The surgical equipment was rigid STORZ hysteroresectope with zero and 30 degree lenses, the loop and roller ball electrodes were the cutting and ablation devices. We used sublingual or vaginal misopristol 200microgram for ripening of cervix 4hours before procedure especially in infertile and nuligravidas. Few cases canceled because of procedure failure like difficulty in cervical dilatation or vision obscure, because of bleeding. The media usually used was saline for diagnostic cases and D/W 5% and glycin for surgical purposes. 

Patients being treated using hysteroscopy were reevaluated at least six months after treatment and success rates were determined. In evaluating treatment results, the patients were included in the study that could be followed up at least six months after treatment. Knowing that this diagnostic and therapeutic method is performed routinely for qualified patients in Alzahra educational therapeutic center and no extra intervention was performed, therefore no specific ethical problem was faced. Patients’ data remained confidential. 

This study has been approved by Tabriz Medical Sciences University ethics committee. The following variables were studied: age, gravidity, parity, and history of previous abortion, number of abortions, duration of the complaints, hospitalization time, contraceptive type, menstruation status, treatment type and complications or failure of diagnosis-treatment. 

**Statistical analysis**

All information entered to computer and used SPSS ver. 15 software for Windows and used One-Way ANOVA test for analysis of data and p<0.05.

## Results

In total 277 females who went under hysteroscopy were divided into three groups: 226 cases with vaginal bleeding, 34 cases of infertility and 17 cases of repetitive abortions. The characteristics of patients are presented in table I. Treatment type in three groups is summarized in table II. 

Complications related to hysteroscopy were not seen in any of the patients except fluid overload in few patients especially with hypotonic solutions. In the group of vaginal bleeding, 134 patients could be followed up. In the group of infertility, 16 patients could be followed up. 5 (33.3%) had become pregnant. the most common indication for hysteroscopy in infertile group was implantation failure and endometrial polyps or adhesions in HSG. In the abortion group 12of the cases could be followed up. 

Septate uterus was the most common findings and septum removal with monopolar resecting electrode was the choice and in some cases the fine semi flexible scissors were the selected instruments especially for thin septum and with normal saline as distending media. Prevention of mid pregnancy wastages was the result but there was one intrauterine death and in some of the patients there was need for cerclage because of ultrasound criteria for concomitant incompetency. There was about 73.5% success rates in managing abnormal bleeding in patients who flowed up with decrease the duration and amount of bleeding with improving hemoglobin after 6 months of treatment.

**Table I:** Demographic parameters of patients

		**Recurrent abortions**	**Infertility**	**Uterine bleeding**	**p-value**
Age (year) (mean±SD)		32.7+6.0	29.6+5.4	41.3+8.3	<0.001
Gravidity (No) (mean±SD)		4.4±1.4	0.8±1.4	3.8±2.5	<0.001
Parity (No) (mean±SD)		1.4±1.6	0.2±0.6	3.3±2.3	<0.001
History of abortion n (%)		7 (100%)	10 (29.4%)	73 (30.9%)	<0.001
Abortion (mean±SD)		3.3±2.0	0.5±1.1	0.6±1.2	<0.001
Admitted duration (mean±SD)		2.9±0.7	2.7±0.9	3.1±1.4	0.377
Contraception [n (%)]					
	No	1 (14.3%)	32 (94.1%)	39 (16.5%)	-
	Tubal ligation	0	1 (2.9%)	72 (30.5%)	
	Vasectomy	0	0	9 (3.8%)	
	Withdrawal	2 (28.6%)	1 (2.9%)	55 (23.3%)	
	Condom	0	0	24 (10.2%)	
	OCP	4 (57.1%)	0	17 (7.2%)	
	IUD	0	0	20 (8.5%)	
Menses [n(%)]					
	Regular	5 (71.4%)	22 (64.7%)	74 (31.6%)	-
	Irregular	1 (14.3%)	11 (32.4%)	140 (59.3%)	
	Amenorrhea	1 (14.3%)	1 (2.9%)	3 (1.3%)	
	Menopause	0	0	19 (8.1%)	

**Table II:** Types of treatment in three groups

	**Recurrent abortions n(%)**	**Infertility n (%)**	**Uterine bleeding n (%)**
Hysteroscopy	14.3%	3 (8.8%)	39 (16.5%)
Hysteroscopy and myomectomy	1 (14.3%)	4 (11.8%)	19 (8.1%)
Hysteroscopy and curettage	2 (28.6%)	6 (17.6%)	88 (37.3%)
Hysteroscopy and polypectomy	0	0	6 (2.5%)
Laparoscopy ovarian drilling	0	1 (2.9%)	0
Hysteroscopy and hysterectomy	0	0	14 (5.9%)
Hysteroscopy and laparoscopy	3 (42.9%)	14 (41.2%)	11 (4.7%)
Hysteroscopy and laparotomy	0	0	3 (1.3)
Hysteroscopy, curettage and polypectomy	0	1 (2.9%)	0
Hysteroscopy, curettage and laparoscopy	0	3 (8.8%)	0
Hysteroscopy, myomectomy and curettage	0	2 (5.9%)	36 (15.3%)
Hysteroscopy, myomectomy and polypectomy	0	0	1 (0.4%)

## Discussion

In this study, we evaluated the diagnostic and therapeutic efficacy of hysteroscopy in managing the common conditions including abnormal uterine bleeding, infertility and abortion.

**a) Abnormal uterine bleeding**

In the group of abnormal uterine bleeding, hysteroscopy was used as the only diagnostic method in 16.5% of the cases. In this group the most common diagnostic therapeutic methods combined with hysteroscopy were curettage, myomectomy, polypectomy and hysterectomy. No complication was observed and in 73.5% of the cases diagnostic and therapeutic measures had led to the treatment of uterine bleeding. Liu et al (2007) in a study on 35 females with abnormal uterine bleeding found that treatments using hysteroscopy were successful in 85.7% of the cases (8). In the study of Engelsen et al (2006) which was carried out on 386 females with abnormal uterine bleeding, hysteroscopy was successful in 83.4% of the cases (9). 

In this study of Loffer et al (2000) which was carried out on the 177 females with abnormal uterine bleeding, hysteroscopy in 95.9% of the cases was successful regarding diagnosis and treatment (). As it can be seen, the results obtained from our study are in accordance with the results obtained from similar studies. “Van Dongen et al (2009) in a study carried out on 21 patients with abnormal uterine bleeding used hysteroscopy as a diagnostic or therapeutic method. The underlying reason was uterine polyp in most of the cases which was treated properly (11)”. “Fuentes et al (2007) in a study carried out on 5103 cases of hysteroscopy due to abnormal uterine bleeding diagnosed and treated 641 cases of endometrial polyps. Only in one case was there a report of seizure and in another case uterine perforation as diagnostic-therapeutic complications (12)”. 

In another study carried out by Fuentes et al (2007), 372 females in the age of menopause with abnormal uterine bleeding were studied. Uterine atrophy, polyp, cancer and hyperplasia were the most common causes of bleeding respectively. In this study, hysteroscopy was introduced as a prime diagnostic and therapeutic method in this group of patients (). In the study of Lasmar et al (2008), 4054 cases of hysteroscopy combined with biopsy were studied. In this study also endometrial polyp was introduced as the most common cause of abnormal uterine bleeding (14) in a study introduced hysteroscopy as the golden standard of treating uterine bleeding caused by uterine myomas (15). Mukhopadhayay et al (2007) in a study compared three methods of transvaginal sonography, hysteroscopy and endometrial biopsy regarding the diagnosis of the reasons causing abnormal uterine bleeding. Finally it should be mentioned that hysteroscopy is the most specific and sensitive diagnostic modality for endometrial polyps (16). 

As it was previously mentioned, most diagnosed patients in this study were performed polypectomy, myomectomy or curettage which is suggestive of the high incidence of benign uterine lesions as underlying bleeding sources. The results obtained from this study are in accordance with the results obtained from similar studies, i.e. hysteroscopy is a safe and efficient diagnostic-therapeutic method in evaluating and treating this group of patients.

**b) Infertility**

In this group, hysteroscopy was used as the only diagnostic methods in 14.3% of the cases. In this group, the most common diagnostic-therapeutic methods combined with hysteroscopy were myomectomy, curettage, polypectomy and adhessiolysis in Ashermans. In a follow-up, pregnancy happened in 33.3% of the cases. In that study of Yanaihara et al (2008) which was carried out on 230 females with infertility, hysteroscopy was reported to be successful in the diagnosis and treatment of uterine polyps (). In that study of Yu et al (2008) which was carried out on 85 females with Asherman syndrome, hysteroscopy led to excellence therapeutic results (). 

In the study of Kaminski et al (2006) which was carried out on 636 females with infertility, hysteroscopy was reported to be a successful diagnostic and therapeutic method in the infertilities with intrauterine pathologies (19). Tiufekchieva (2006) reported a 100% success in using hysteroscopy as a diagnostic and therapeutic method in the infertilities related to intrauterine pathologies (). Radestad and Bins, (2009), in a study, showed that benign uterine pathologies are one of the important reasons of infertility in females which could be treated safely and efficiently using hysteroscopy (). 

Boudhraa et al (2009) evaluated 200 females with infertility using hysteroscopy. 75% of the cases had abnormal uterine findings including polyp, fibrosis and various malformations (22). Godinjak and Idrizbegovic, (2008) in a study suggested hysteroscopy as a routine diagnostic and therapeutic modality in the females with infertility (). Based on the results obtained from the previously mentioned studies and the findings from the present study, this diagnostic-therapeutic method is suggested in the females with infertility.

**c) Abortion**

In the abortion group, hysteroscopy was used as the only diagnostic method in 14.3% of the cases. In this group, Laparoscopy, curettage and myomectomy were the most common associated diagnostic-therapeutic measures respectively. Filho et al (2006), in two studies, demonstrated that this method is efficient in the diagnosis of abortion causes and in the treatment of possible cases (24, 25). Dendrinos et al (2008) studied 48 infertile females. In 52% of the cases hysteroscopy was normal. In the rest of the cases, myomas, adhesion, polyp and structural disorders were reported. In this study, hysteroscopy was introduced as a safe and efficient diagnostic-therapeutic method in these patients (). In this study of Ventolini et al (2004), 23 patients with repetitive abortions were studied. In 60.9% of the cases, there were no abnormal findings (27). 

In the rest of the cases structural uterine disorders, myomas, adhesion were reported. Pregnancy ratio was 29% after treatment (). Weiss et al (2005), in a study, concluded that hysteroscopy is indicated in every female with two abortions (28). The results obtained from our study are accordance with the similar studies however the number of patients with repetitive abortions is low in the present study. Hysteroscopy was used as the only diagnostic method in the groups with abnormal uterine bleeding, infertility and abortion in 16.5%, 8.8% and 14.3% of the cases. In the group of abnormal uterine bleeding, curettage, myomectomy, polypectomy, hysterectomy, were the most common diagnostic-therapeutic approaches combined with hysteroscopy respectively. In the group of infertility, laparoscopy, myomectomy, curettage, polypectomy were the most common diagnostic-therapeutic approaches combined with hysteroscopy respectively. 

In the group with abortion, laparoscopy, curettage and myomectomy were the most common diagnostic-therapeutic approaches combined with hysteroscopy respectively. No major complication was reported in any of the patients except fluid overload in operative cases with hypotonic distending Medias. In the follow-up of patients, bleeding cessation and pregnancy was reported in two groups with abnormal uterine bleeding and infertility in 73.5% and 33.3% of the cases respectively. Based on the results obtained from the present study, hysteroscopy is a safe and efficient method in diagnosis and treatment of the causes leading to abnormal uterine bleeding, infertility and repetitive abortions and therefore it should be used as a less invasive method in the management of these patients. 

Further prospective studies with higher sample volumes are needed to reach more precise results on the efficacy of hysteroscopy in the patients with repetitive abortions that can replace a major complicated laparotomy and uterine incision with a minimally invasive procedure outpatient. With the technological progress each technical procedures are improving and hysteroscopy with safer bipolar electrodes and mini endoscopes with flexible scopes is going to substitute these methods with conventional ones for intra uterine pathologies. Use of misoprostol for ripening of the cervix as for uterine evacuation was very useful for dilatation of cervix for operative rigid hysteroresectoscopy especially in nulligravidas (29). It must be mentioned that less operative complications by any approach demand more expertise, and to improve endoscopic surgery, it must be included in the educational curriculum of gynecological residents (30). 
